# Is Personality Disorder Madness? A Qualitative Study of the perceptions of Medical Students in Somaliland

**DOI:** 10.1192/j.eurpsy.2023.2381

**Published:** 2023-07-19

**Authors:** H. R. Arisna, J. Handuleh, K. Bhui, T. Lee

**Affiliations:** 1 Psychiatry, East London NHS Foundation Trust; 2 the Centre for Understanding of Personality, DeanCross Personality Disorder Service, London, United Kingdom; 3 Psychiatry, St Paul Hospital Millenium Medical College, Addis Ababa, Ethiopia; 4 Dept of Psychiatry & Nuffield Department of Primary Care Health Sciences Medical Sciences Division, University of Oxford, Oxford, United Kingdom

## Abstract

**Introduction:**

Patients with borderline personality disorder are often a challenge to the mental health system. Psychiatrists see people with BPD as manipulative, difficult to manage, annoying, unlikely to arouse sympathy, clinicians hold negative attitude towards personality disorder.

As the next generation of doctors, medical students’ perception of patients with personality disorder (PD) is critical.

Yet a systematic review of the literature shows this has not been studied.

**Objectives:**

The study aims to identify :

1) the understanding and perception of medical students about PD

2) factors that may relate to this knowledge and perception.

**Methods:**

A focus group discussion (FGD) was conducted with eight medical students in their sixth year at Amoud University, Somaliland.

A case vignette of a patient with typical Borderline PD symptoms was presented to stimulate discussion. Barts Explanatory Model Inventory (BEMI) was used to explore the issue.

The FGD was conducted via MS teams, recorded, transcribed, translated and thematically analysed

**Results:**

The Medical students showed reasonably accurate knowledge regarding Borderline PD, recognising features of unstable mood, impulsiveness, and emptiness. Of note half the participants believed religious intervention would be helpful *“I believe in Islam. So,basically so to some degree it could be managed in certain religious centers”.* Importantly, medical students, when asked to divest of their professional identity, and to describe their personal views associated PD with madness.

**Conclusions:**

The views of PD as ‘madness’ and that religious intervention has a role have important implications for training and service development.

The importance of a culturally sensitive training to Medical students regarding PD to match local cultural and religious views, and consideration of development of health services which are sensitive to religious practice is highlighted.

We recommend including social and cultural implications in the training of medical students to better prepare them for the complexity of managing PD.

**Disclosure of Interest:**

None Declared

